# Metformin induces cell cycle arrest, apoptosis and autophagy through ROS/JNK signaling pathway in human osteosarcoma

**DOI:** 10.7150/ijbs.33787

**Published:** 2020-01-01

**Authors:** Bo Li, Pingting Zhou, Kehan Xu, Tianrui Chen, Jian Jiao, Haifeng Wei, Xinghai Yang, Wei Xu, Wei Wan, Jianru Xiao

**Affiliations:** 1 Department of Orthopedic Oncology, Changzheng Hospital, Second Military Medical University, Shanghai, China; 2 Department of Radiation Oncology, Shanghai Ninth People's Hospital, Shanghai Jiaotong University School of Medicine, Shanghai, China

**Keywords:** Osteosarcoma, Metformin, Apoptosis, Autophagy, ROS, JNK

## Abstract

Metformin, an ancient drug commonly used for treating type II diabetes, has been associated to anti-cancer capacity in a variety of developing cancers, though the mechanism remains elusive. Here, we aimed to examine the inhibitory effect of metformin in osteosarcoma. Herein, we demonstrated that metformin treatment blocked proliferation progression by causing accumulation of G2/M phase in U2OS and 143B cells. Furthermore, metformin treatment triggered programmed cell death process in osteosarcoma cell lines. Further research indicated the induction of apoptosis and autophagy triggered by metformin could remarkably attenuate after the treatment of ROS scavenger NAC and JNK inhibitor SP600125. Additionally, our results showed that NAC-suppressed JNK/c-Jun signaling pathway could have been activated through metformin treatment. Lastly, metformin could inhibit osteosarcoma growth under safe dose *in vivo*. Thus, we propose that metformin could induce cell cycle arrest as well as programmed cell death, including apoptosis and autophagy, through ROS-dependent JNK/c-Jun cascade in human osteosarcoma. This metformin-induced pathway provides further insights into its antitumor potential molecular mechanism and illuminates potential cancer targets for osteosarcoma.

## Introduction

Osteosarcoma (OS), the most commonly malignant bone cancer with a tendency to the metaphysis of long bones, has the highest morbidity in children, adolescents, and young adults 1-5. Despite the development in neoadjuvant chemotherapy plus surgery, the 5-year survival frequency for patients is still less than 70% and 30% in cases with localized disease and metastatic disease, respectively^6^, ^7^. Unfortunately, it is still a maze in the development of better strategies and new methods, which highlights the importance of more effective therapies for OS treatment.

Metformin is the most commonly used drug for treating type II diabetes with safe effects of insulin resistance reduction and blood glucose decrease 8, 9. It also exhibits a number of attributes that make it appealing for repurposing as an anti-cancer therapy 10. Metformin has been associated to have anti-cancer capacity in developing cancers, particularly in melanoma and pancreatic cancer cells 11-13. It is widely known that adenosine monophosphate-activated protein kinase (AMPK), participates in sensing energy in mitochondrion, could been activated after the energetic inhibition by metformin treatment 13-16. However, several new studies have specified that AMPK is not the only pathway for metformin's beneficial effects, requiring other major metformin downstream effectors of mitochondria 17-19. A recent report indicated that lifespan of *C. elegans* was increased and growth inhibition in cancer cells was induced after metformin treatment were activated by a key transcriptional target, *ACAD10*, which indicates AMPK pathway is not indispensable for metformin mediated mechanism of tumor suppression 20. Although some previous reports demonstrated that treatment with metformin might prevent the development of OS cells, the underlying molecular mechanisms for suppressing the growth of human OS is still subject to ongoing investigation.

Normal cell proliferation depends on a complete and effective cell cycle under the regulation of several critical checkpoint kinases, such as cyclin-dependent kinases (CDKs) as well as proteins which can inhibit CDK 21, 22. The checkpoint of G2 phase prevents entry into mitosis with DNA damage, causing cell cycle arrest as well as inducing senescence or apoptosis endpoints.

In malignance cells, cell cycle imbalance is an early step for tumor development. Many cytotoxic agents were found to play an important part in the arrest of G2/M cell cycle 23, 24. Apoptosis (type I) and autophagy (type II) are two main types of programmed cell death (PCD) for cells. Apoptosis, the most common defined type of PCD, is controlled by intracellular and/or extracellular signals and described by stereotypical morphological alterations such as nuclear fragmentation and condensation, membrane blebbing, cell shrinkage and apoptotic body development. During the past three decades, signaling pathways involved in apoptosis have been widely focused in multiple tumor cells. Autophagy has been identified as a “self-eating” process highlighted by the vesicular sequestration and degradation of cytoplasmic components 25. Autophagy is activated and regulated by cell stressors, such as hypoxia, reactive oxygen species (ROS), osmotic stress and infection. The potential molecular mechanisms between two PCD types remain elusive. Under some circumstances, autophagy can induce cell apoptosis, while in others; it participates in pathway of cell surviving to suppress apoptosis 26, 27. Therefore, a further understanding of the interaction of two PCD types remains to be determined.

ROS, known as an inducer or mediator for the trigger of the mitogen-activated protein kinase (MAPK) family members, has a substantial effect in multiple usual biochemical roles and irregular pathological progressions 28. In cancer research, increasing studies suggest that substantial quantity of ROS is involved in apoptosis and autophagy. c-Jun-N-terminal kinase (JNK) of MAPK family is crucial for many cellular progresses, such as apoptosis and autophagy 29, 30. Hence, targeted activation on the ROS/JNK signaling cascade might be beneficial in treating malignant tumors.

Here, we found the inhibitory role of metformin in human OS cells *in vitro* as well as* in vivo*. Furthermore, we investigated the underlying molecular pathways by which metformin induced cell cycle arrest, cell death as well as autophagy regulated by the ROS/JNK signaling cascade.

## Materials and Methods

### Cell lines

The human OS cell lines (143B, U2OS) analyzed in this study were kindly provided by the Cell Bank of China Academy of Sciences (Shanghai, China). The cells were incubated with Dulbecco's modified Eagle medium (DMEM) comprising 10% fetal bovine serum (FBS, UT, USA) at 37℃ with 5% carbon dioxide.

### Antibodies and reagents

1,1-Dimethylbiguanide hydrochloride (metformin) (#D150959) was obtained from Sigma Aldrich, then was dissolved in distilled water at 1M concentration and finally was stored at -20°C before use. 3-MA (M9281) was purchased from Sigma-Aldrich. SP600125 (#1460) was purchased from Selleckchem (Houston, TX, USA). NAC, Hoechst staining as well as JC-1 were bought from Beyotime Institute of Biotechnology (Shanghai, China). The antibodies to cleaved-PARP (#5652), cleaved-caspase3 (#9661), Bcl-2 (#15071), LC3II/I (#12743), p62 (#8025), Beclin-1 (#3495), JNK (#9252), phospho-JNK (#4668), c-Jun (#9165), phospho-c-Jun (#3270), Cyclin D1 (#2978), and secondary antibodies (anti-rabbit and anti-mouse) were bought from Cell Signaling Technology. The antibody to GAPDH (#I121209) was bought from TransGen.

### Cell viability assay

143B and U2OS cells (10000 cells/well) were sowed in 96-well plates for 24 h. Next, they were treated with metformin at different dosages (0, 5, 10, 20, 40 mM) for 24-72h. Cell viability was assessed by Cell Counting Kit-8 (CCK-8) (Dojindo, Japan). Spectrophotometer was used to measure the absorbance at 450 nm and the cell growth curve was described according to the absorbance values.

### Colony-formation assay

To examine the capacity changes of single cells to form a colony, 143B and U2OS cells (2000 cells/well) were seeded and incubated at different concentrations of metformin to form colonies for 14 days. After 4% paraformaldehyde was used to fix the colonies, 0.1% crystal violet staining was done for 15 minutes at room temperature (RT). The images were taken and quantified under the microscope. The colonies including >50 cells were added up microscopically.

### Cell cycle analysis

After 143B and U2OS cells were treated with multiple dosages of metformin for 24 h. Following this, they were digested, centrifuged, washed, and fixed for 30 min using chilled ethanol. Then they were treated with propidium iodide (PI) for an hour at RT in dark and observed by flow cytometer (Beckham, USA).

### Morphological apoptosis

To observe apoptosis phenomenon, the Hoechst 33342 staining assay was performed. 5 × 10^4^ cells per well were incubated in 6-well plates overnight and treated with 0, 20 mM metformin for 24 hours. Next, Hoechst 33342 solution was used to analyze cell morphology as per the supplier's protocol. Morphological changes of the nucleus were examined by fluorescence microscope.

### Mitochondrial membrane potential (MMP) assay

The alteration of MMP was examined by the JC-1 Assay kit (Beyotime, Shanghai, China). 5 × 10^5^ cells were incubated in 6-well plates overnight at 37 °C in a 5% carbon dioxide incubator and metformin was used to treat 143B and U2OS cells in a dosage dependent manner for 24h. Next day, supernatants were removed from culture dishes and cells were treated with JC-1 staining solution for 20 min at 37 °C in a 5% CO_2_ incubator, and then examined by flow cytometry (Beckham, USA).

### Apoptosis analysis by flow cytometry

To measure apoptotic cell death induced by metformin treatment, Annexin-V-FITC Apoptosis Detection Kit (BD Biosciences, USA) was employed. 143B and U2OS cells after metformin treatment were digested, washed thrice with chilled PBS and then resuspended in binding buffer. Next, they were incubated in FITC-labeled Annexin V as well as PI for 15 minutes at RT in dark and evaluated by flow cytometer.

### ROS assay

ROS were detected with probe DCFH-DA (Beyotime Biotechnologies, Beijing, China). Cells pretreated with metformin at different concentrations were incubated with DCFH-DA according to the manufacturer's introduction. Then, ROS was observed by fluorescence microscope (Olympus, Japan) and analyzed by flow cytometer (Beckham, USA).

### GFP-LC3 puncta assay

Firstly, GFP-LC3 lentivirus was used to transfect 143B cells. And fluorescent puncta of autophagosomes formation presented intercellular autophagy. After transfected for 24 h, 143B cells were treated with 0, 10 mM or 20 mM metformin for another 24 h. Confocal microscope (Leica, Germany) was used to obtain images.

### Western blotting analysis

Radioimmune precipitation assay (RIPA) buffer was used to lyse the cells and centrifuged (12, 000 g, 10 min, 4°C). The similar amount of proteins was first loaded on SDS-PAGE for electrophoresis and later on transferred to PVDF membranes. Now the membranes were blocked using 5% nonfat milk for an hour at RT. Next, the blots were immunoblotted overnight at 4 °C using specific primary antibodies such as LC3, p62, beclin-1, cleaved-caspase-3, Bcl-2 and cleaved-PARP (Cell Signaling Technology, USA). Next day, the blots were incubated for an hour at RT with polyclonal secondary antibodies before being visualized by using a chemiluminescence detection kit (Milipore, USA).

### Immunohistochemical analysis

Paraffin-embedded tumor tissue of tumor xenografts was sectioned and immunohistochemically stained for PCNA, p-JNK, and cleaved caspase-3 using a kit from Dako (Copenhagen, Denmark). Following antibodies were bought from Santa Cruz: PCNA (sc-390003, 1:50, 4°C overnight), p-JNK (sc-390003, 1:50, 4°C overnight), and cleaved caspase-3 antibody (sc-390003, 1:50, 4 °C overnight). Vectastain Elite DAB KIT was bought from Vector Laboratories (CA, USA).

### Tumor xenograft

Male BALB/c nude mice of four to six-week-old (Shanghai Slac Laboratory Animal Co. Ltd.) were raised in a standard laboratory environment with food and water. An injection of a total of 2 × 10^6^ 143B cells suspended in 100 μl chilled PBS was given into the medullary cavity of mice tibia. The animals were randomly allocated into different groups: intraperitoneal injections of 200 mg/kg of DMSO or 200 mg/kg of metformin every other day. Tumor size was documented every 3 days by the formula: tumor volume = 0.5 × L× W^2^. Mice were killed after 15 days of metformin treatment. The tumors were detached, weighted and fixed using 10% formalin for additional examination. All experimentations were in compliant with the National Institutes of Health Animal Use Guidelines and permitted by the Laboratory Animal Center of Second Military Medical University.

### Statistical analysis

All the cell culture assays were performed in triplicates for at least three times. GraphPad Prism 7.0 software (CA, USA) was used for data analysis. Obtained data were represented as means with SDS, unless indicated otherwise. Student's t-test or one-way ANOVA was employed for analyzing the variance amongst diverse groups. P value of <0.05 was considered statistically significant.

## Results

### Metformin prevents proliferation and triggers cell cycle arrest in OS cells

**Figure 1:**
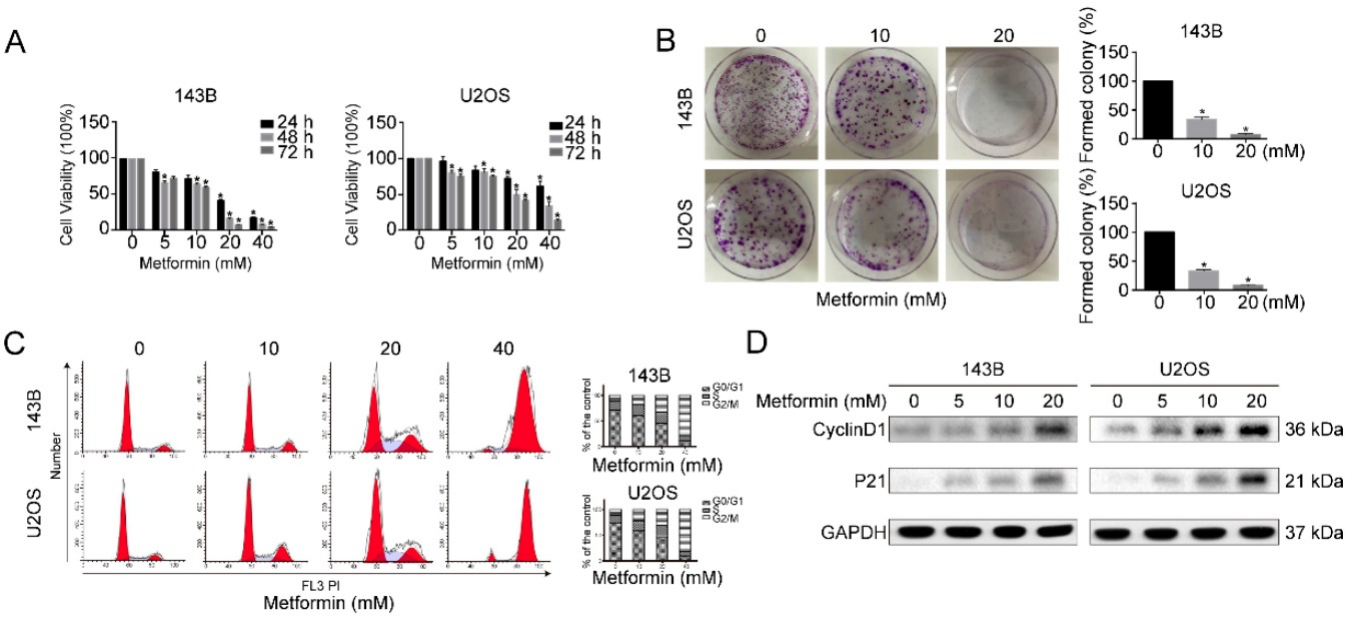
** Metformin inhibited the growth of OS cells. (A)** For 24 h-72h, both 143B and U2OS cells were incubated with 5-40 mM metformin followed by quantifying cell number with CCK-8 assay. **(B)** The ability of OS cells to form clones after metformin and relative control treatment assessed by colony formation assay. **(C)** Metformin caused G2/M cell cycle arrest as revealed by flow cytometry. **(D)** Western blotting analysis of Cyclin D1 and P21 expression in the indicated groups. GAPDH was considered as control. *P < 0.05.

To examine the anti-proliferative functions of metformin on OS and human OS cells (143B and U2OS) were treated with diverse dosages of metformin either for 24 or 48 or 72 hours. Analysis was done by CCK-8 assay. The cell proliferative capacity was significantly inhibited after metformin treatment in both dose and time-dependent fashion (Fig. 1A). Additionally, colony-formation assays showed that colonies formation was significantly decreased by the treatment with metformin (Fig. 1B). This suggested that metformin prevents the cell viability of OS cells.

To confirm the relation between the growth inhibition and cell cycle arrest, next we analyzed the function of metformin on the progression of cell cycle. Compared with untreated controls, G2/M accumulation and a downtrend in G0/G1 peak was observed in metformin treated 143B and U2OS cells at given concentrations after 48 h (Fig. 1C). Furthermore, western blotting results showed that cell cycle-related proteins Cyclin D1 as well as P21 were clearly up-regulated by metformin treatment (Fig. 1D). Taken together, these findings suggest that metformin encouraged cell cycle arrest at G2/M phase, by leading to regulation of the proteins related to cell cycle.

### Metformin induces apoptosis in OS cells

**Figure 2:**
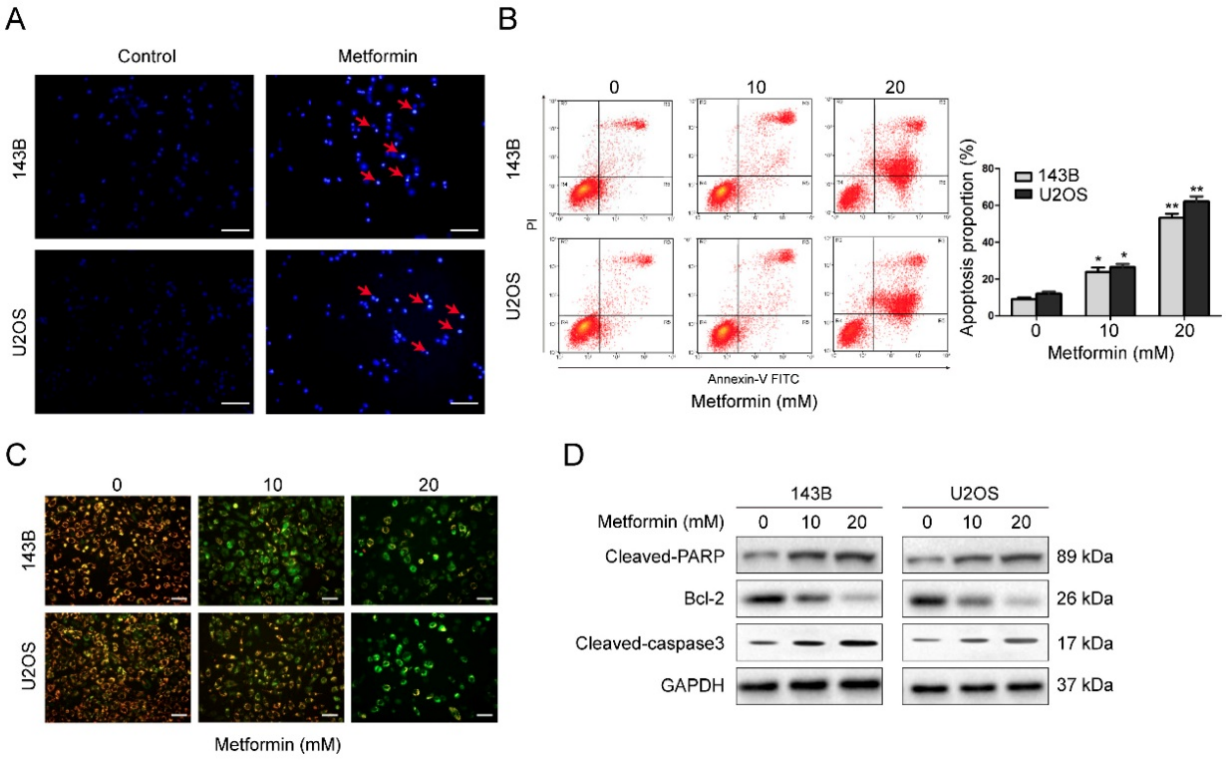
** Metformin induced apoptosis of OS cells. (A)** Representative images of apoptotic nuclear morphology changes evaluated by Hoechst 33342 staining. Condensation of chromatin as well as fragmentation of nuclear is indicated by arrows. Scale bars = 50 μm. **(B)** OS cells were used for flow cytometric analysis to evaluate the percentage of apoptosis. **(C)** Alterations in the mitochondrial membrane after the treatment with metformin were observed by JC-1 staining. Scale bars = 20 μm.** (D)** Western blot examination of cleaved PARP, cleaved-caspase 3 and Bcl-2 expression in the indicated OS cells. GAPDH was used as control. **P < 0.01, *P < 0.05.

Apoptosis commonly is related to cell cycle arrest. Therefore, we explored the apoptosis function of metformin in OS cells. Hoechst 33258 was used to stain apoptotic nucleus of OS cells. After incubation with 20 mM metformin for 48 h, both 143B as well as U2OS cells showed apoptotic characteristics, such as shrinkage of the cells, condensation of chromatin as well as fragmentation of the nuclei (Fig. 2A). This was performed by flow cytometer. Results demonstrated that after treated with given concentrations of metformin, the percentage of cell apoptosis was significantly increased (Fig. 2B).

MMP (*ΔΨm*) loss is a crucial step during apoptotic process. Next, we analyzed *ΔΨm* by probe JC-1 to investigate whether mitochondrial was involved in the apoptotic induction by metformin. After treatment with 20 mM metformin for 48 h, the change of fluorescence color from red to green was obviously observed in a dosage-dependent manner (Fig. 2C), showing that metformin lead to a depletion of *ΔΨm* in OS cells. Next, to further understand the process by which metformin causes apoptosis, western blotting was conducted. Exposure of the OS cells to metformin in a dosage-dependent manner caused a stimulation of cleavage caspase-3 and PARP. Additionally, there was a reduction of the expression of Bcl-2 (Fig. 2D). Thus, the above data suggest that metformin promoted cell apoptosis in OS cells.

### Metformin induces autophagy of OS cells

**Figure 3:**
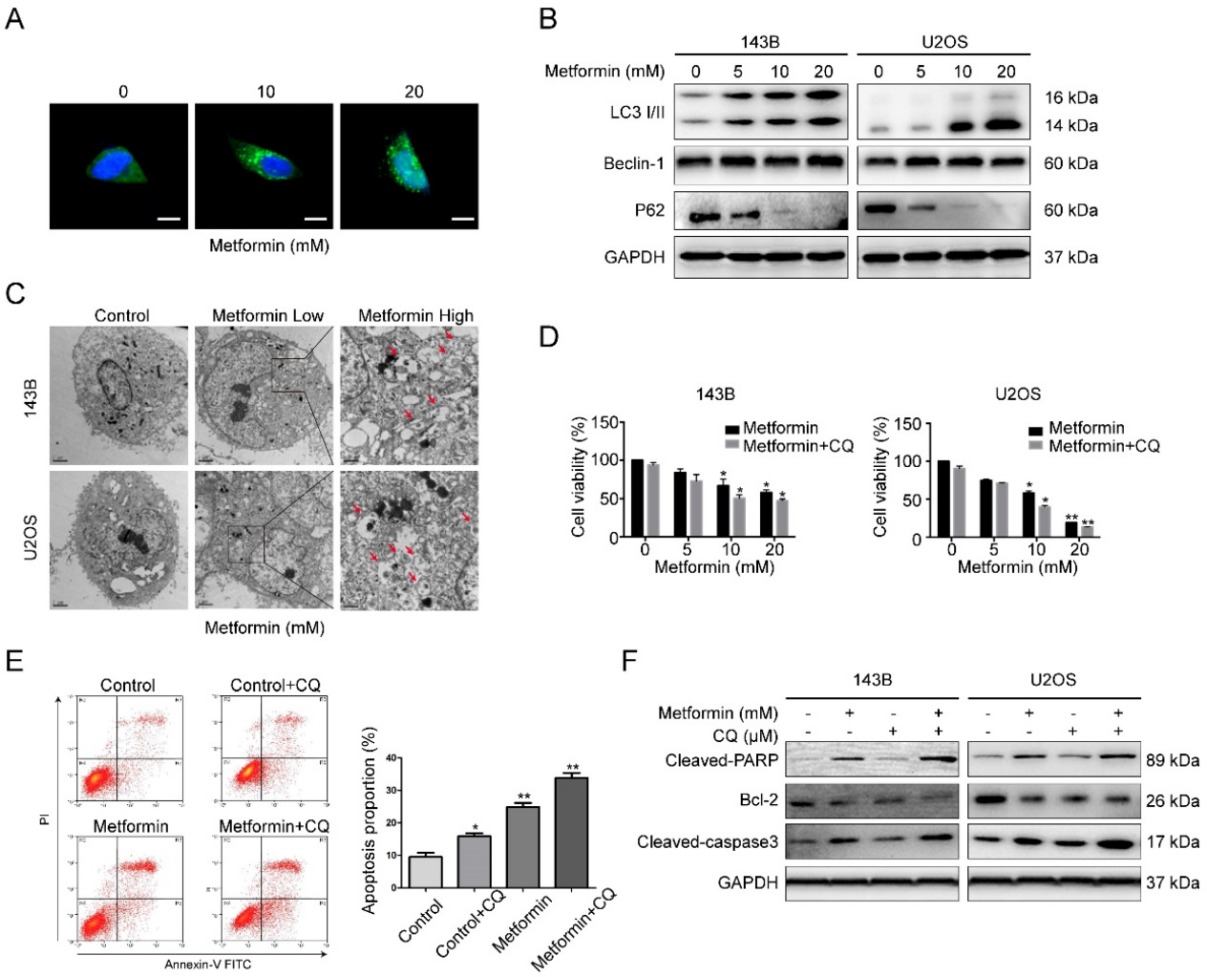
** Metformin caused autophagy of OS cells and inhibited autophagy enhanced metformin-induced apoptosis. (A)** Representative images of OS cells stably expressing GFP-LC3 in metformin treatment and control groups. Scale bars = 10 μm. **(B)** Western blotting analysis of LC3 I/II, Beclin-1 as well as P62 expression in the indicated OS cells. **(C)**Transmission electron microscopy images of OS cells that were used to detect autophagosomes. Control, Low 10,000X; High 20,000X.** (D)** Cell number were quantified using CCK-8 assay following blockade of autophagy by pharmacological inhibitor CQ and metformin treatment. **(E)** The apoptotic OS cells were investigated by flow cytometric assay following the treatment with metformin with or without CQ. (**F)** Apoptosis-associated proteins were studied after the treatment with metformin in presence or absence of CQ. **P < 0.01, *P < 0.05.

The above data demonstrated the participation of apoptosis in metformin-induced cell death; however, it was unclear whether autophagy, which could contribute to cell death, was involved. Autophagy is known as a degradation process of protein, during which the constituents in cells are digested in lysosome. Firstly, GFP-LC3 lentivirus was transfected in 143B cells to detect the fluorescent puncta formation of autophagosomes. After exposure to metformin for 48 h, green puncta formation presented an obvious increase in a dosage-dependent manner (Fig. 3A). Next, western blotting assay was used to test the several marker proteins of autophagy. It showed that metformin treatment upregulated the level of LC3B-II, p62 and Beclin-1 in OS cells in a dosage-dependent manner (Fig. 3B). Autophagosomes captured by electron microscopy is featured as a direct evidence of autophagy. In our study, it was shown that autophagic vacuoles obviously increased in the cytoplasm of metformin-treated cells compared to control cells through transmission electron microscopy (TEM) (Fig. 3C).

Since autophagy has both positive and negative roles for therapeutic purpose in tumor, representing the effects of preventing or promoting apoptotic cell death. We then used the autophagy blocker chloroquine (CQ, 20 μM) to prevent metformin-induced autophagy in 143B cell. Flow cytometric analysis results presented in Fig. 3D specified that administration with CQ might enhance the inhibitory function of metformin on cell proliferation. Furthermore, treatment with CQ strengthened metformin apoptotic effects (Fig. 3E-F). All the above mentioned outcomes indicated that metformin triggered autophagy of OS cells in a dosage-dependent manner. The autophagy triggered by metformin may be anti-apoptotic.

### Metformin stimulates JNK/c-Jun pathway by inducing ROS generation in OS cells

**Figure 4:**
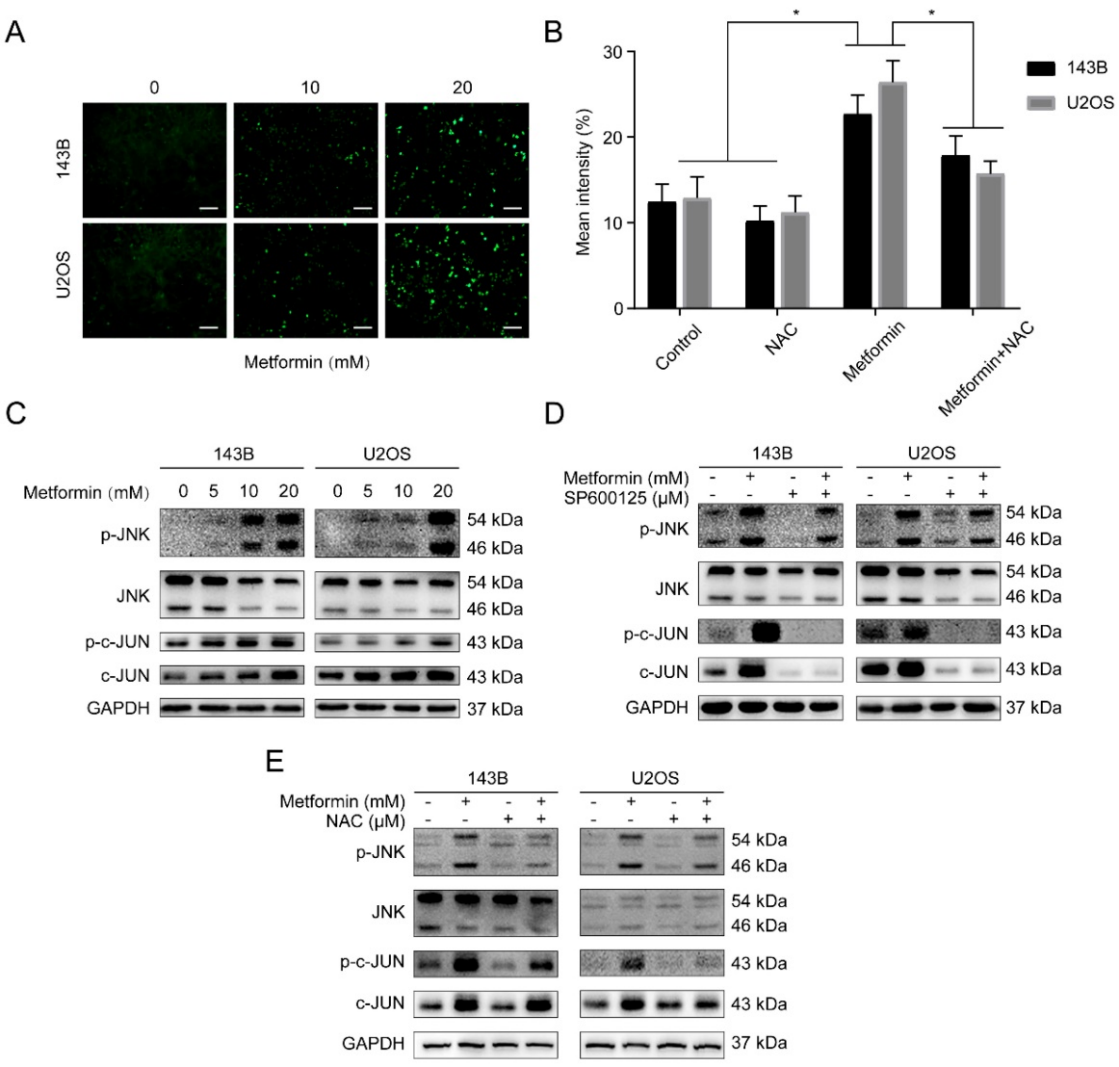
** Metformin stimulated JNK/c-Jun pathway by inducing ROS generation in OS cells. (A)** Intracellular ROS levels were measured by fluorescence microscope with DCFH-DA staining and **(B)** analyzed by flow cytometer. Scale bars = 50 μm.** (C)** Immunoblots of p-JNK, JNK, p-c-Jun and c-Jun protein expressions in OS cells.** (D-E)** Cells were incubated with metformin and were pretreated with SP600125 and NAC. Related protein expression was quantified with the help of western blot technique. *P < 0.05.

Reactive oxygen species (ROS) produced either from the action of NADPH oxidase (NOX) or from the mitochondrial respiratory chain, was reported to share crucial functions in progress of apoptosis and autophagy 31. Besides, the loss of *ΔΨm* in metformin-treated OS cells was revealed by flow cytometry assay. This guided us to believe that metformin may trigger the accumulation of ROS from mitochondria. Fig. 4A demonstrated that compare to the control group, OS cells treated with metformin showed a significant increase of ROS. The antioxidant N-acetyl cysteine (NAC), worked as ROS scavenger, was applied to further analyze the elevation of ROS. We then performed the DCFH-DA flow cytometry assay showing that exposure of metformin in OS cells augmented ROS levels, that could be markedly suppressed by NAC (5 mM, 2 h) (Fig. 4B). Accumulating evidence showed that ROS puts the finger on the button of the JNK/c-Jun signaling cascade 29, 30. Then the function of metformin on JNK/c-Jun pathway was investigated. The results showed that metformin increased phosphorylation of JNK and c-Jun in OS cells in a dosage-dependent manner (Fig. 4C). Additionally, following the 2-hour pretreatment with JNK inhibitor SP600125 (30 μM) and NAC (5 mM), the above phosphorylation effect was reversed in OS cells (Fig. 4D-E). Collectively, we demonstrated that metformin could activate the ROS/JNK signaling pathway.

### Metformin induces apoptosis and autophagy through activating ROS/JNK signaling cascade in OS cells

**Figure 5:**
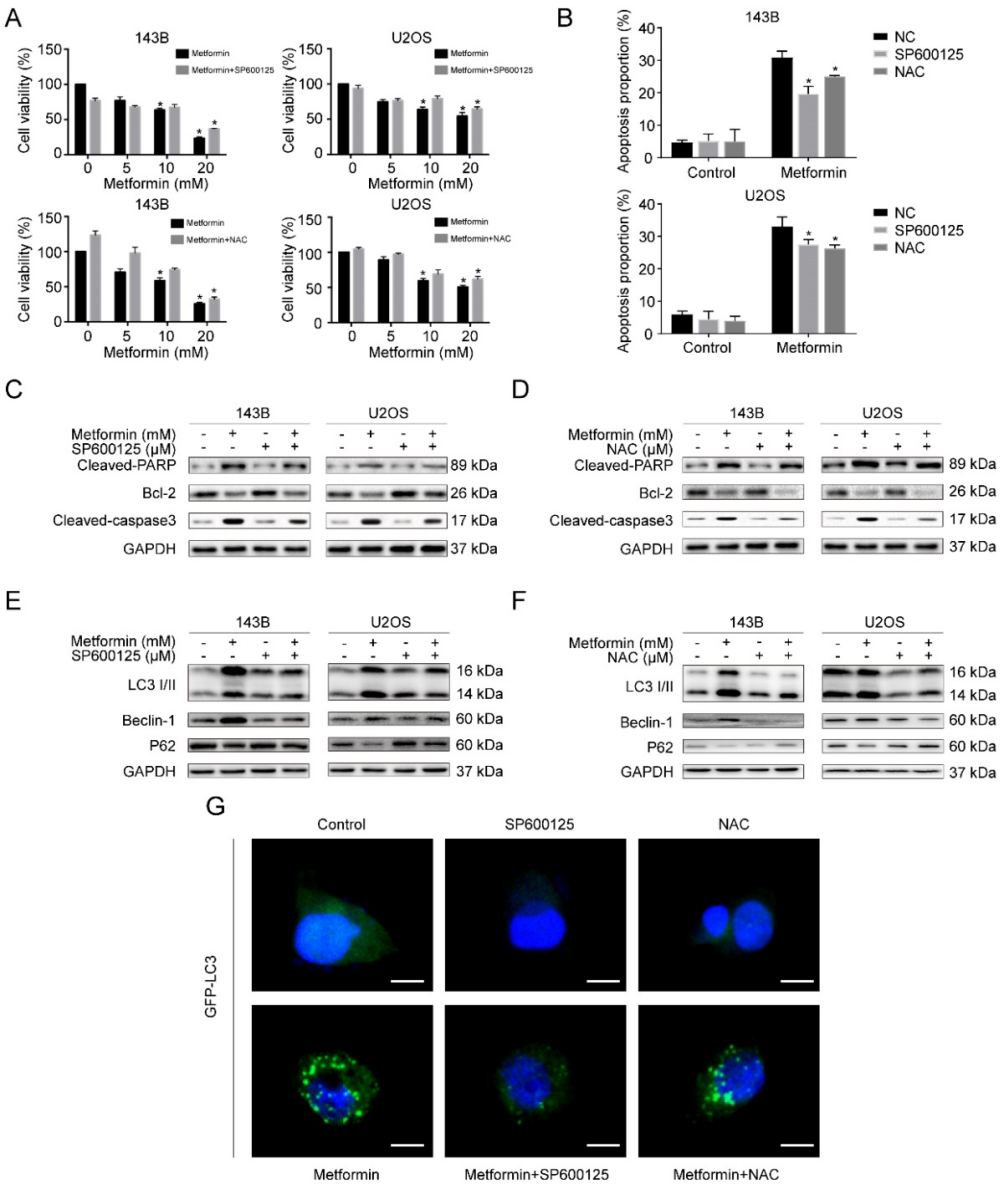
** Metformin caused apoptosis and autophagy through activating ROS/JNK cascade in OS cells. (A)** CCK-8 assay was conducted to evaluated cell viability. **(B)** The apoptotic cells were evaluated by flow cytometry. **(C-D)** The apoptosis-related proteins levels were determined by western blotting. GAPDH was considered as a control. **(E-F)** The expression levels of autophagy-related proteins were determined by western blotting. GAPDH was considered as a control. **(G)** Representative images of OS cells steadily articulating GFP-LC3. OS cells were pretreated with or without SP600125 or NAC and then incubated with metformin and control. Scale bars = 10 μm. *P < 0.05.

We then investigated whether metformin caused apoptosis and autophagy by causing the accumulation of ROS along with the activation of JNK in human OS cells. Firstly, we pre-treated OS cells with SP600125 and NAC for 2h, respectively. After that, we treated cells with metformin for additional 48h. Significantly, CCK-8 analysis exhibited that SP600125 and NAC could weaken the inhibitory effect of cell viability induced due to metformin (Fig. 5A). Further, we demonstrated that the metformin-induced apoptosis effect was notably reduced after pre-treating using SP600125 and NAC by flow cytometric analysis (Fig. 5B). In addition, consistent with the above results, western blotting results revealed that the two inhibitors altered the proteins related to cell-death (Fig. 5C, 5D). Afterward, we reversely examined the participation of ROS accumulation and JNK pathway stimulation in autophagy that was induced by metformin. The outcomes presented that the autophagy-related proteins, LC3-II, Beclin-1 and p62, were decreased after pre-treating with SP600125 and NAC (Fig. 5E and 5F), and the number of GFP-LC3 fluorescence intensity shared a consistent trend (Fig. 5G). Taken together, the activation of ROS/JNK signaling cascade had a role in metformin-induced apoptosis as well as autophagy.

### Metformin prevents growth of OS in xenograft tumors

**Figure 6:**
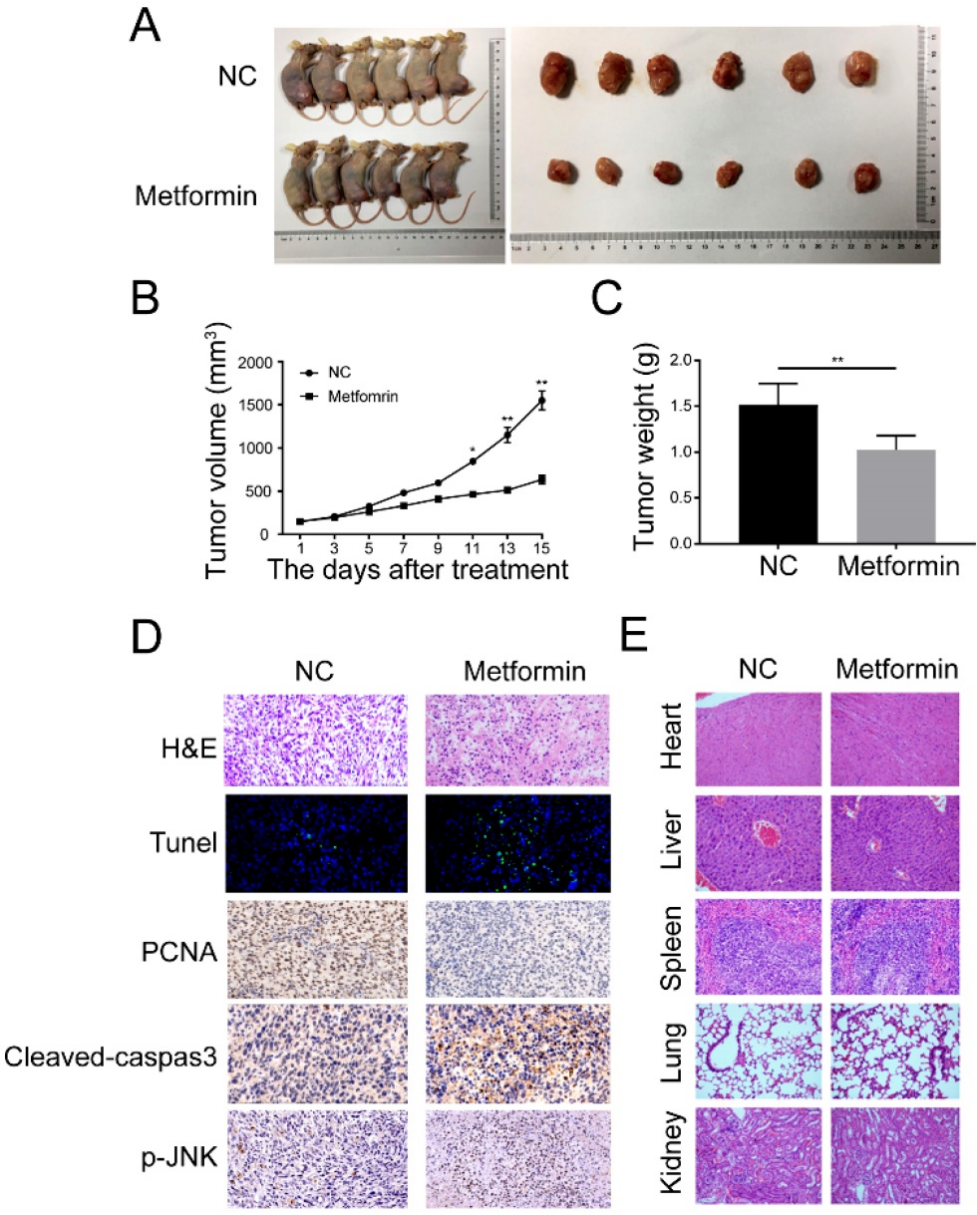
** Metformin repressed the growth and caused the apoptosis of OS cells *in vivo*. (A)** Representative images of BALB/c-nude mice following the injections of 143B cells vehicle or metformin group. **(B)** Tumor volume was recorded every 3 days after metformin treatment. **(C)** The tumor was removed and weighted after all mice were killed. **(D)** The expression of Tunel, PCNA, cleaved-caspase-3, and p-JNK were observed by immunohistochemistry. **(E)** H&E staining was done to assess the histological aspects of major organ. **P < 0.01, *P < 0.05.

For *in vivo* studies, we used the BALB/c nude mice to build a xenograft OS model through injecting 143B cells into tibial. The mice were arbitrarily allocated into either control or metformin group 10 days after injection. It showed that metformin remarkably reduced the growth of OS without significant loss of the body weight in 200mg/Kg metformin treatment group (Fig. 6A and 6B). In addition, metformin treatment caused the decrease of mean tumor weight (Fig. 6C). Immunohistochemistry results indicated that the amount of terminal dUTP nick end labeling (TUNEL)-positive cells was augmented, as well as the levels of cleaved caspase-3 and p-JNK in mean areas, while the expression of PCNA was decreased (Fig. 6D). Hematoxylin and eosin (H&E) staining exhibited that compare to the control group, no obvious main organ-associated injuriousness was detected in the metformin-treated group (Fig. 6E). Together, all these data indicated that metformin exhibited anti-osteosarcoma potential at a safe dose *in vivo*.

## Discussion

Metformin has been used for nearly 60 years at low cost and is the most widely prescribed medication for treating type 2 diabetes 8, 32. It also exhibits potential attributes that make it appealing for benefit in cancer prevention and treatment 10, 33. Metformin has showed anti-tumor capacity in multiple malignant tumors, such as melanoma and pancreatic cancer. The mitochondrion is widely accepted as the main target of metformin, and inhibition by metformin results in mitochondrial energetic stress, which leads to the stimulation of the energy sensor AMPK 15, 18. However, accumulating evidences indicate that AMPK pathway is not a must in the metformin's anti-tumor benefit function, which invokes further investigation on other metformin effectors downstream of mitochondrion 17, 19, 20. Here we have detected that metformin can inhibit cell proliferation, trigger cell cycle arrest and induce two types of PCD in OS cells. Further experiments indicated that metformin induces PCD progresses by activating ROS/JNK cascade *in vitro* as well as *in vivo*.

Cell cycle dysregulation is a characteristic of multiple tumor development. G2 checkpoint, preventing cells from undergoing mitosis when DNA damage becomes incontrollable, may be a repair chance for self-healing 24, 34. According to the results of flow cytometry, metformin increased the G2/M phase along with a reduction of the G0/G1 proportion in cell cycle of OS cells. In addition, western blot assay indicated that metformin induced an increase the protein level of Cyclin D1, which has an essential role in regulating the transition of G2/M-phase. Additional results showed that augmented expression level of P21 was observed under metformin treatment and it has a crucial role in hindering the stimulation of the complex Cdk1/Cyclin D1. These results suggested that metformin could induce the G2/M phase accumulation and caused the corresponding phase arrest in OS cells. However, the underlying mechanism in this situation still remains elusive and needs to be further explored.

Cell apoptosis could be induced by cell cycle arrest and is reported to allow cells to be a part of the tumor development and treatment response 21, 35. Multiple cancer treatments such as chemotherapy, radiotherapy, immunotherapy and gene therapy, have put the finger on the button of apoptosis signal transduction pathway 4, 36. Accumulated evidence has indicated that mainly two signal pathway participate in the regulation of apoptosis, including intrinsic and extrinsic way 37, 38. Mitochondria have a vital role in the intrinsic pathway of apoptosis 39. The results in this study also showed that MMP was significantly decreased after the treatment of metformin, which indicated that metformin could induce the depolarization of mitochondria in OS cells. Current studies have proved that cytochrome c first releases from the intramembrane of mitochondria following the depolarization of mitochondrial membrane, then activate the cytosolic caspases 40, 41. The other pathway of cell apoptosis is extrinsic way, which includes the ligand interaction with death receptors that include Fas/CD95 as well as the protease caspase family proteins, leading to the apoptotic cascade 26. In this study, metformin caused cell death in OS cells by activating caspase 3 and PARP. In addition, immunohistochemical analysis and TUNEL experiment revealed a substantial escalation in the proportion of cell apoptosis in metformin-treated xenograft OS tissue. Our results indicated that apoptosis might be elicited by activating the extrinsic and intrinsic pathways after metformin treatment.

Beside apoptosis, autophagy, which also has a vital role in regulating cell death, has been widely concerned. Throughout the course of autophagy, materials and organelles in cells get seized into autophagosomes, then decomposed and digested in the lysosome 25, 42. Accumulated data indicates that autophagy behaves as a two-edged sword in cancer, providing protection or causing damage for cells 26, 27, 43, 44. In our study, we showed that metformin could induce autophagy with the results that the number of autophagic vesicles increased along with the enhanced expression levels of LC3B-II, p62 and Beclin-1. Our results also indicated that CQ, one of autophagy inhibitors, could strengthen the power of metformin-induced apoptosis, which means autophagy induced by metformin may support survival during the death of OS cells.

A high level of ROS after the metformin treatment in OS cells was found during the research of apoptosis and autophagy phenomenon. Normal metabolism of oxygen produces physiological level of ROS, which may play roles in promoting cell proliferation and survival 45. However, excessive level of ROS could contribute to apoptosis and autophagy following the injury of cellular components 28. Our current results indicated that metformin triggered a dramatic increase of ROS generation, while the effect of metformin on cell proliferation, apoptosis and autophagy could remarkably reverse under the treatment of NAC, the ROS scavenger. Our data specified that metformin may trigger the stimulation of apoptosis and autophagy by induction of high level of ROS.

Mitochondria are the main place for the generation of ROS and it is also one of the main target organelles of metformin 20, 46. Previous studies have showed that mitochondrial inhibition could cause energetic stress, leading to the activation of the energy sensor, AMPK 47, 48. However, growing evidence indicated that AMPK is dispensable for metformin's beneficial effects, invoking other metformin-induced pathway downstream of mitochondria 17-20. Recently, accumulated studies indicated that the JNK signaling pathway could transduce oxidative stress signals and induce cell apoptosis and autophagy under various stress stimuli 26, 30, 49, 50. Is there a possible connection between metformin effects and JNK pathway? In this study, we have reported that a remarkable augmentation of JNK and c-Jun phosphorylation was detected after the metformin treatment. The stimulation of JNK pathway had a role in the regulation of metformin-induced cell death as well as autophagy, which was also tested by the pretreatment with SP600125, a JNK inhibitor. Furthermore, we found that the accumulation of ROS could trigger the stimulation of JNK and c-Jun pathway, whereas pretreatment with NAC could attenuate the phosphorylation of JNK and c-Jun induced by metformin. Taken together, these results showed that metformin could induce apoptosis and autophagy by triggering ROS-dependent JNK/c-Jun cascade without involvement of AMPK.

In conclusion, our results showed that metformin could significantly prevent tumor production by inducing cell cycle phase arrest, and cell PCD progress in human osteosarcoma. Furthermore, metformin induced two types of PCD through activating ROS-dependent JNK/c-Jun signaling pathway. Additionally, autophagy inhibition with CQ enhanced metformin-induced apoptosis, suggesting metformin-induced autophagy played a protective role in OS cells. Therefore, we initially identified metformin antitumor functions on human OS without any involvement of AMPK pathway. Taken together, these findings not only indicated the probable antitumor mechanism of metformin, but offer an alternate approach of combining metformin with autophagy inhibitor together for human OS treatment as well.
