# Metformin induces cell cycle arrest, apoptosis and autophagy through ROS/JNK signaling pathway in human osteosarcoma: Erratum

**DOI:** 10.7150/ijbs.74954

**Published:** 2022-07-08

**Authors:** Bo Li, Pingting Zhou, Kehan Xu, Tianrui Chen, Jian Jiao, Haifeng Wei, Xinghai Yang, Wei Xu, Wei Wan, Jianru Xiao

**Affiliations:** 1 Department of Orthopedic Oncology, Changzheng Hospital, Second Military Medical University, Shanghai, China; 2 Department of Radiation Oncology, Shanghai Ninth People's Hospital, Shanghai Jiaotong University School of Medicine, Shanghai, China

The original version of our paper contained an error. In Figure 4, the western blot images of JNK for 143B and U2OS in Figure 4C were mistakenly presented in Figure 4D. This error was caused by unintentionally covering the correct images during figure assembly. The correct version of the Figure 4D appears below.

All authors agree to the erratum and confirm that the corrections made in this erratum do not affect the original conclusions. The authors apologize for any inconvenience that the errors may have caused.

## Figures and Tables

**Figure 4:**
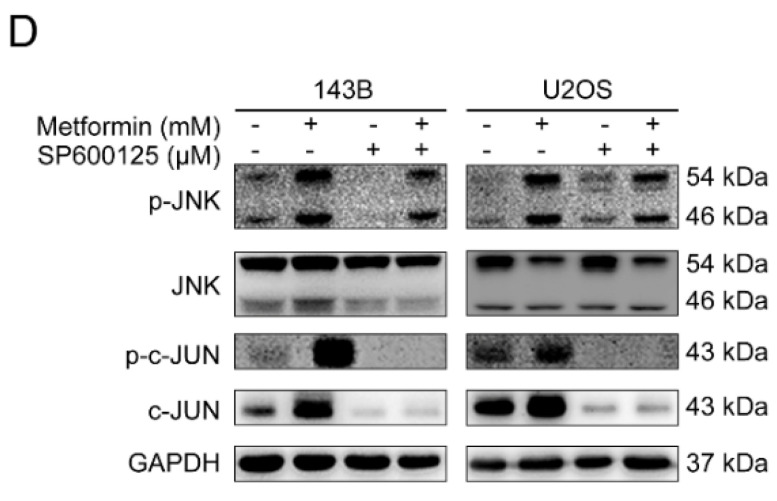
** (D)** OS cells were incubated with metformin and were pretreated with SP600125.Western blot analysis for p-JNK, JNK, p-c-Jun and c-Jun protein in 143B and U2OS cells.

